# Small-Angle Scattering as a Structural Probe for Nucleic Acid Nanoparticles (NANPs) in a Dynamic Solution Environment

**DOI:** 10.3390/nano9050681

**Published:** 2019-05-02

**Authors:** Ryan C. Oliver, Lewis A. Rolband, Alanna M. Hutchinson-Lundy, Kirill A. Afonin, Joanna K. Krueger

**Affiliations:** 1 Neutron Scattering Division, Oak Ridge National Laboratory, Oak Ridge, TN 37830, USA; ryanoliver5683@gmail.com; 2 UNC Charlotte Chemistry Department, Charlotte, NC 28223, USA; lrolband@uncc.edu (L.A.R.); ahutch27@uncc.edu (A.M.H.-L.); kafonin@uncc.edu (K.A.A.)

**Keywords:** small-angle X-ray scattering, small-angle neutron scattering, contrast variation, nucleic acid nanoparticle, structural characterization

## Abstract

Nucleic acid-based technologies are an emerging research focus area for pharmacological and biological studies because they are biocompatible and can be designed to produce a variety of scaffolds at the nanometer scale. The use of nucleic acids (ribonucleic acid (RNA) and/or deoxyribonucleic acid (DNA)) as building materials in programming the assemblies and their further functionalization has recently established a new exciting field of RNA and DNA nanotechnology, which have both already produced a variety of different functional nanostructures and nanodevices. It is evident that the resultant architectures require detailed structural and functional characterization and that a variety of technical approaches must be employed to promote the development of the emerging fields. Small-angle X-ray and neutron scattering (SAS) are structural characterization techniques that are well placed to determine the conformation of nucleic acid nanoparticles (NANPs) under varying solution conditions, thus allowing for the optimization of their design. SAS experiments provide information on the overall shapes and particle dimensions of macromolecules and are ideal for following conformational changes of the molecular ensemble as it behaves in solution. In addition, the inherent differences in the neutron scattering of nucleic acids, lipids, and proteins, as well as the different neutron scattering properties of the isotopes of hydrogen, combined with the ability to uniformly label biological macromolecules with deuterium, allow one to characterize the conformations and relative dispositions of the individual components within an assembly of biomolecules. This article will review the application of SAS methods and provide a summary of their successful utilization in the emerging field of NANP technology to date, as well as share our vision on its use in complementing a broad suite of structural characterization tools with some simulated results that have never been shared before.

## 1. Introduction

Nucleic acid-based nanoparticles (NANPs) [1,2,3,4,5,6,7,8,9,10,11,12,13] and other nucleic acid-based nanodevices [14,15,16,17] are an emerging research focus area in pharmacological and biological studies. NANPs can be designed and manipulated to produce a variety of different functionalized nanostructured scaffolds; the novel resultant structures require detailed characterization prior to further biomedical transition and in vivo studies [7,18,19,20,21,22]. Conventional characterization techniques include the routinely used analysis of NANPs by native-PAGE (non-denaturing PolyAcrylamide Gel Electrophoresis) [23], dynamic light scattering (DLS) [4], atomic force microscopy (AFM) [24], and more sophisticated methods employing cryogenic-electron microscopy (cryo-EM) [25], nuclear magnetic resonance (NMR) [26], and X-ray crystallography [27] (see Table 1 for a comparison of the advantages and limitations of these structural characterization techniques). However, none of the aforementioned techniques allows for direct visualization of large (>100 kDa) three-dimensional NANPs in solution, and they often require working with high concentrations of NANPs.

Small-angle scattering (SAS) is a structural characterization technique that is well placed to determine the conformation of NANPs under varying solution conditions, which will allow for optimization of their design and pipe-line production. SAS experiments yield information on the overall shapes and electron (or nuclear) density distribution within macromolecules in solution (for additional primers on this technique, see [33,34,35,36,37]). In addition, the inherent differences in the neutron scattering of nucleic acids, lipids, and proteins allow for the characterization of the conformations and relative dispositions of the individual components of an assembly of biomolecules using methods of contrast variation or solvent matching. Also, due to the different neutron scattering properties of the isotopes of hydrogen the neutron scattering contrast can be enhanced by labeling one component within a macromolecular assembly with deuterium. An example application would include using contrast variation methods to examine the overall shape of NANPs that have been functionalized with short interfering RNAs (siRNAs), ribozymes, aptamers, proteins, or other small molecules (for a review of functionalized nanoparticles see [38,39]. Conformational changes within the NANP itself as a result of direct or indirect fusion with these therapeutically relevant molecules could then be observed independently. Small-angle neutron scattering (SANS) combined with contrast variation or contrast matching methods would allow for detection of the conformation of each of the individual components within the resultant NANP assembly, as well as the distance between their centers of mass. A structural basis for understanding the resultant functionalized NANPs will be essential to guarantee precise control over the composition and stoichiometry of therapeutic modules for simultaneous delivery into diseased cells and eventually for their successful transitions to in vivo preclinical studies [7,11,18,19,20,40]. Certainly, the direct visualization of various NANPs and NA multi-stranded assemblies can be, and has been, achieved with AFM [41] and cryo-EM [7], as mentioned previously. However, the resolution of these techniques is currently limited by the size (with smaller NPs < 20 nm being preferred), shape, and composition of the nanoparticles. Also, neither of these techniques addresses the complicated, dynamic environment of the particle in solution. Therefore, to gain additional information about the structure of NANPs and to understand more completely the structure-to-function relationship, thus possibly enhancing its functionality, several techniques must be combined. This article will review the application of small-angle X-ray and neutron scattering methods and provide a summary of their successful utilization in the emerging field of NANP technology to date. Importantly, we share our vision for how it may be used in the near future to add to and complement a broad suite of structural characterization tools with some guidance and the feasibility of the proposed applications by including some simulated results that have never been shared before.

## 2. Discussion

A model for the structure of nucleic acids was initially proposed by Watson and Crick in 1953. Interpretation of the X-ray diffraction patterns from meticulously prepared 2D deoxyribonucleic acid (DNA) crystals recorded by Rosalind Franklin provided the key to understanding this structure. Since then, X-ray diffraction has played a vital role in further discoveries of numerous types of nucleic acid structures. An excellent review of the applications of various synchrotron-based spectroscopic techniques, including small-angle X-ray scattering (SAXS), has been recently published by Yi Lu and his research group [42]. High-resolution techniques such as X-ray crystallography have the capability to provide atomistic structural views. However, SAS techniques can be applied to molecules in solution and can give insights into systems in which inherent flexibility, which may cause problems for crystallization, is in fact essential for its proper function. SAS was first described for biomolecules (proteins) in the late 1940s [43] and has been widely used for several decades to solve problems requiring an understanding of the nanoscale phenomena. SAS techniques in fact depend on the same physics as the corresponding larger angle scattering methods (X-ray or neutron diffraction), but reveal larger, more global structures due to the inverse relationship between length scale and scattering angle; refer to Glatter and Kratky [44] for an excellent textbook describing the physics of SAS.

In lieu of a crystal, X-ray scattering from nucleic acid samples in solution can provide essential structural information on the time-averaged ensemble structure. Information about the size, shape, compactness, and molecular weight of the scattering molecules are readily obtained from the scattering data. Beginning in the late 1980s, as the methods of analysis and image reconstruction technologies became more accessible and sophisticated (see the ATSAS software package [45]), so too did the functional insights and applications. Thanks to advances in computational capabilities and instrumentation, particularly with the increased flux available now at synchrotron sources, SAS has developed into a powerful structural tool that complements and enhances other structural information to provide a more complete understanding of the structure-function relationship. For example, Wang and co-workers used SAS to describe an unusual topological structure that the HIV-1 (human immunodeficiency virus) uses to recognize its own messenger ribonucleic acid (mRNA) [46]. SANS and contrast variation techniques are ideally suited to examining the conformational changes within the protein and its nucleic acid binding partner upon complexation with one another. Recently, Sonntag et al. [47] demonstrated the power of contrast variation and SANS in resolving ambiguities and improving the interpretation of complementary SAXS and NMR data on a ternary protein-RNA complex involved in alternate splicing.

Of importance in extending these SAS technologies to study NANPs specifically, SAS provides not only information on the sizes and shapes of particles but also information on the internal structures of disordered and partially disordered systems. Rambo and Tainer [48,49] have improved and tested the SAS computational tools and technologies specifically for applications to the inherently flexible nucleic acid and related structures. Their SAXS results have discovered and demonstrated that conformational variation is a general functional feature of macromolecules. Importantly, SAS can tolerate a variety of measurement conditions, thus allowing rapid comparison of the effects of environmental changes on the detected structural properties. Moreover, extraction of meaningful 3D details from 1D scattering data via molecular modeling techniques has become increasingly sophisticated [50,51], allowing for the development of experimentally constrained structural models that can be further interpreted or constrained by other types of structural knowledge on the system being studied (for recent reviews see [35,52]). Indeed, a major concern in interpreting resultant SAS-based models is that there may be several structures that produce similar scattering patterns. One must always keep in mind that these models represent the time-averaged ensemble, which could include a mixture of dynamic conformations and/or intermolecular interactions. For this reason, complementary data from other structural techniques is essential to proper interpretation.

*Small-Angle Scattering Methodology:* Light scattering, in general, is useful for studying the state of association or conformation of biological macromolecules in solution [53]. Both static (elastic) and dynamic (quasi-elastic) light scattering techniques are generally easy to perform and can be done on solutions with relatively low concentrations of analyte. The static light scattering (SLS) experiment monitors the total light scattering intensity averaged over time and can provide information on the “apparent” molecular weight (M_app_) and the radius of gyration (R_g_) of the macromolecule in solution. Dynamic light scattering (DLS) experiments monitor fluctuations in the intensity of light scattered by small volume elements in solution, which are directly related to the Brownian motion of the solutes, thereby providing information on the hydrodynamic radius (R_H_), which also can be related to an apparent molecular weight. In either case, light scattering techniques can be used as an initial probe of the NANP conformations to monitor aggregation or conformational changes in varying solution environments. Determining particle size and shape, however, requires a light source with much smaller wavelengths, such as X-rays or neutrons.

**Figure 1:**
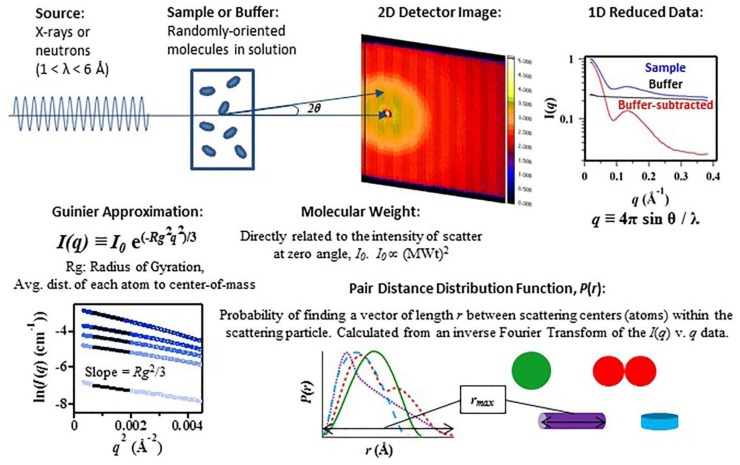
Typical small-angle X-ray and neutron scattering (SAS) experimental set-up and data analysis. I(q) is the intensity of the scattered light as a function of momentum scattering vector, q, as defined above. I(0) is the intensity of the scatter at zero angle and is directly proportional to the square of the molecular weight of the biomolecule (MWt)^2^. R_g_ is the biomolecule’s Radius of Gyration, and is defined as the average distance of each scattering center, atom, from the center-of-mass. P(r) is the pair distance distribution function, calculated as an inverse Fourier Transform of the scattering data and representative of the probability of finding a vector of length r between the atoms within the biomolecule.

X-ray and neutron SAS represents a major tool for obtaining global information on the size and shape of folding intermediates of RNA molecules in solution, since it provides quantitative characterization of mixtures by measuring the radius of gyration and maximum linear dimension of the molecules to ~1–10 nm resolution. Typical experimental set-up and analysis is shown in Figure 1. A sample containing randomly oriented molecules in solution is placed in an X-ray or neutron beam with wavelengths between 1–6 Å. The coherent scattering, *I*(*Q*), from a homogeneous solution of monodisperse particles can be expressed mathematically as:

*I*(*Q*) = 〈 | ∫ [*ρ* (*r*) − *ρs*]•exp(−*iQ*•*r*) *dr* | 2 〉 (1)

The integration is taken over the volume of the particle and 〈 〉 denotes the average over all particle orientations. *Q* is the momentum transfer or scattering vector and can be expressed as 4π(*sin*θ)/λ, where θ is half the scattering angle and λ is the wavelength of the scattered radiation. *ρ* (*r*) and *ρs* are the scattering length densities for the particle and solvent, respectively. Structural information is derived from a measurement of the intensity of the scattered X-ray (I(Q)) as a function of scattering angle (Q). Analysis of these data is accomplished initially with a Guinier approximation by fitting the data in the low Q region (where Q⋅Rg < 1.3). This approximation can be done for globular or for rod-like particles and yields a direct estimation of the molecule’s R_g_ or cross-sectional radius (R_c_), respectively. For well-folded samples, a Kratky plot can be used to estimate the hydrated volume, or Porod volume. Comparative changes in a Kratky plot can reveal flexibility, unfolding, or a conformational change.

More detailed structural information may be obtained from analysis of the pair-distance distribution function, P(r). An inverse Fourier transformation of the scattering data yields the probable distribution of atom-pair distances (r) weighted by the product of their scattering powers, and is typically represented as a 1-dimensional P(r) versus r profile. For well-behaved samples, the P(r) will approach zero at the maximum linear dimension, d_max_, of the scattering particle. The zeroth and second moments of the P(r) give forward scatter, I_0_, and radius of gyration, R_g_, respectively. The forward scatter, I_0_, is directly proportional to the molecular weight squared of the scattering molecule and thus is very sensitive to changes in the size of the scattering particle due to, for example, complex formation, specific oligomerization, or aggregation. P(r) is sensitive to the symmetry of the scattering particle and to the relationships between domains or repeating structures. This effect is demonstrated in Figure 1, which shows the P(r) functions for various one- and two- domain structures of uniform scattering density. It is worth noting how the asymmetry of the P(r) function increases with the asymmetry of the shape of the object. Determining the 3-dimensional shape that gives rise to a measured SAS (intensity versus Q) profile is recognized as an ‘underdetermined’ problem (as a result of rotational averaging of the scattered intensity arising from the random orientation of molecules in solution). Nonetheless, molecular modeling of these data can be highly informative, particularly if the models are interpreted by utilizing other known structural constraints [54]. One interesting approach to assessing the ambiguity in SAS profiles has been reported [55]. An accepted practice is to generate multiple solutions using Monte Carlo-based minimization methods and simple constraints, such as connectivity and compactness, and then to evaluate the variability and range of potential solutions. The software for completing this type of analysis is available in the popular ATSAS analysis package [45].

**Figure 2:**
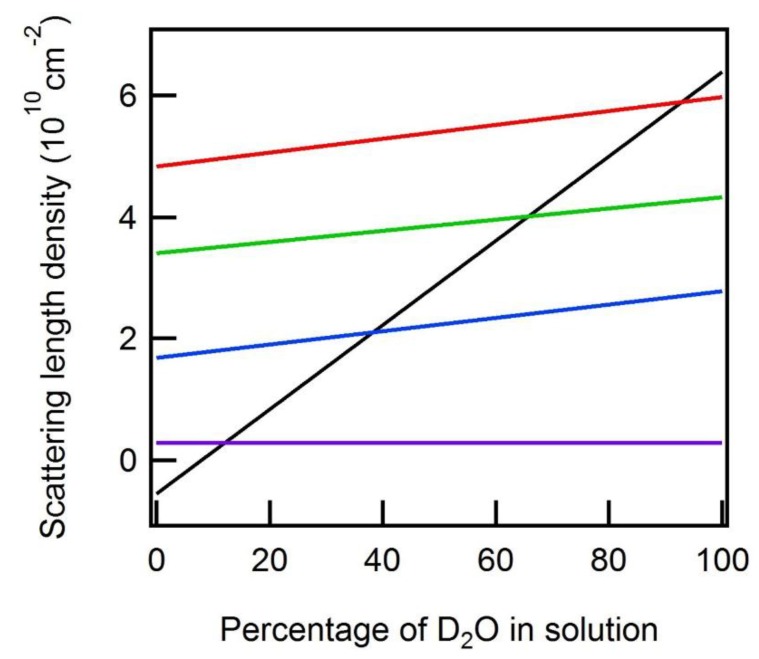
Scattering length densities of various biological macromolecules plotted as a function of percent D_2_O in the solvent: a hydrogenated protein (blue), a deuterated protein with 65% of the non-exchangeable protons replaced by deuterium (red), messenger ribonucleic acid (mRNA) (green), and lipid (purple). The black line corresponds to the scattering length density of the background solution. The “match” point of these macromolecules (circled) is found at the percent D_2_O where the scattering light density (SLD) of the solvent equals that of the molecule.

*Neutron (SANS) Methodology:* Examination of the individual component structures within the context of larger macromolecular assemblies (NA:protein or NA:lipid:protein structures, for instance) can be achieved by collecting neutron scattering data on the complexes while varying the solvent contrast (for a recent review see [56]). Scattering length densities (SLDs) are calculated by summing the scattering amplitudes of each atom within a volume and dividing by that volume. From Equation (1) it can be readily seen that the intensity of the scattering from a particle in solution depends upon the difference in scattering density between the particle and the solvent, i.e., its “contrast”. The SLD of a particle is a function of its elemental composition and the associated atomic scattering lengths (specifically the coherent scattering lengths, b_coh_), which are a measure of the strength of the interaction of an X-ray or neutron with an atom. The fact that hydrogen and its isotope deuterium have dramatically different scattering lengths (b_coh_ = −3.74 × 10^−15^ m and 6.67 × 10^−15^ m, respectively) empowers a neutron scattering contrast variation technique for structural biology. The fraction of D_2_O substitution for H_2_O in aqueous buffers provides a continuous spectrum of values for the solvent’s SLD. The true utility of being able to change the SLD of the solvent relative to that of the scattering particle becomes evident when working with structures composed of materials which have different SLDs, such as proteins, lipids, and NAs. These various biomolecules have an inherently dissimilar elemental composition and thus different average scattering lengths, so each will be ‘visible’ (or ‘invisible’) at unique solvent contrasts. Furthermore, the production of deuterium-enriched biological materials makes possible the reconstitution of multi-component structures with selectively deuterated subunits. Example SLDs of various biological macromolecules, including examples involving deuterium-labeled material, are shown in Figure 2 as a function of the H_2_O/D_2_O mixture in the background solution.

Of particular interest in this plot are the intersections where the scattering length density of the solvent (black line) matches that of the various biomolecules. At these points (referred to as contrast match points), the contrast between the molecule and background (solvent), and therefore the measured intensity of that molecule, is zero. The measured scattering intensity I(Q) from a multi-subunit assembly containing a subunit(s) with a solvent contrast-matched SLD would only reflect the remaining subunit(s), which have a nonzero contrast. The result is structural information on individual subunit components within the macromolecular complex. This particular kind of experiment is known as a contrast-matching experiment. An extension of the contrast-matching experiment is a contrast variation experiment. In a contrast variation experiment, the total scattering of the complex is measured at several solvent contrasts (fractions of D_2_O) and then mathematically extrapolated to yield the scattering profile of the individual components.

*SANS Applications*: A classic set of examples for the application of SANS with contrast variation involves the study of various ribosomes. The earliest studies probed the internal structure of the 30S ribosome [57,58,59]. Contrast-variation methods were used to determine the relative distances between subunits in this multi-subunit complex. Ultimately, this research led to a structural model for the disposition of these subunits in space [60]. These early studies were followed by subsequent studies of the larger 50S and 70S ribosomes. A map of the distribution of protein and RNA within the 50S ribosome from *Escherichia coli* was generated using SANS with contrast variation data and shape restoration by spherical harmonics [61,62,63].

**Figure 3:**
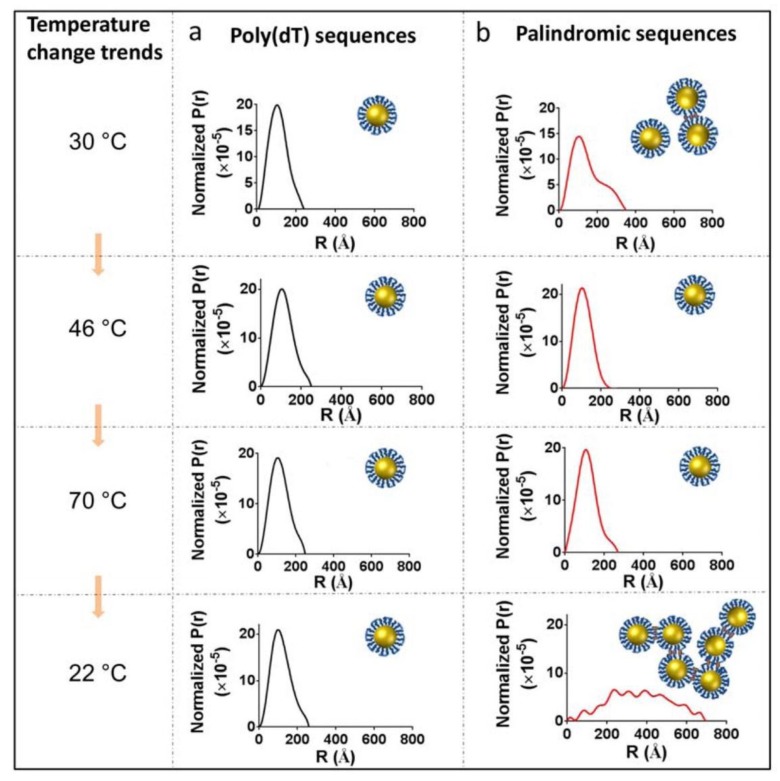
The pair distribution function P(r) is shown for two DNA-capped nanoparticles, T15 (**a**) and T7−8 (**b**) conjugates computed at various temperatures (30 °C, 46 °C, 70 °C, and 22 °C) in 0.5 M salt buffer. Insets are the scheme of temperature effect on poly(dT) sequenced deoxyribosenucleic acid (DNA) and palindromic sequenced DNA. Reprinted (adapted) with permission from (Yang, W.; et al. Probing Soft Corona Structures of DNA-Capped Nanoparticles by Small Angle Neutron Scattering. *J. Phys. Chem. C* 2015, *119*, 18773–18778). Copyright (2015) American Chemical Society.

More recently, SANS has demonstrated the structural influence that ionic strength and temperature have on the corona structures found in DNA-capped gold nanoparticles [64] (Figure 3). These data will assist in customizing tailor-made corona structures for designer materials and devices. X-ray data has provided information on the inorganic cores of these nanoparticles but the complementary neutron data has expanded the structural scope, revealing the 15-mer DNA capped corona structures and the formation of ionic strength- and temperature-dependent aggregate species.

*SAXS Applications:* The structures of small fragments of functional RNAs have been successfully solved using SAXS and confirmed by other techniques [65,66]. This approach allows for an investigation of the influence of the size, composition, and shape of functionalized NANPs on their ability to be delivered to diseased cells and to further their functional efficiency. Structural models built from the solid-state, i.e., X-ray crystal diffraction or cryo-EM, can be used to generate an expected scattering curve, which can then be directly compared with the measured solution scattering data to detect differences in the solution state of the particle. One ultimate goal might be to utilize SAXS under varying solvent conditions (e.g., ionic strength, pH, or binding partners, etc.) in order to gain a better understanding of structure-to-function relationships in various synthetic and natural RNAs. These results would assist in refining computational-experimental protocols for functional RNA nanoparticle pipeline production. Additionally, time-resolved methods using bright synchrotron sources could provide kinetic insights into their assembly. These methods have been successfully used to provide kinetic data on ribozyme folding [67], transfer ribonucleic acid (tRNA) assembly [68], and riboswitches [69,70], so precedents exist for using them to examine the assembly dynamics of NANPs.

**Figure 4:**
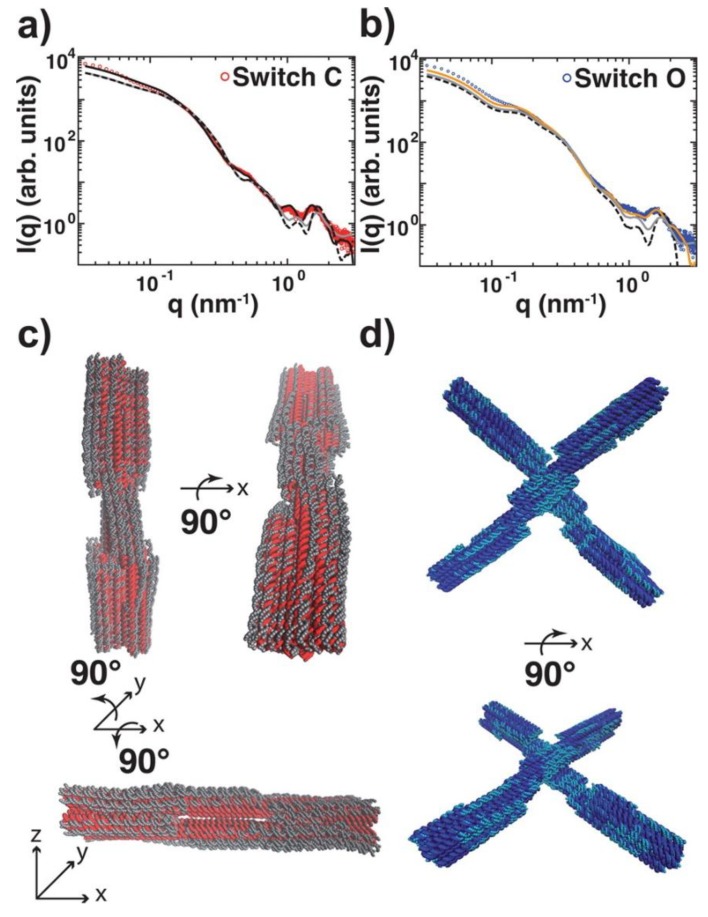
(**a**) and (**b**) depict the mode-based refinement of a DNA switch structure against small-angle X-ray scattering (SAXS) data. (**a**) Experimental SAXS data for the DNA switch in the closed, linear conformation and (**b**) the open, X-shaped conformation are shown as red and blue circles, respectively, against the scattering profile predicted by preliminary models in CanDo (dashed black lines) and CRYSOL software (gray lines) [73]. The preliminary structures of the (**c**) open and (**d**) closed switch conformations are shown as red and blue cylinders with the refined structures shown as gray and light-blue orbs, respectively. Reprinted (adapted) with permission from Bruetzel, L.K.; et al. Conformational Changes and Flexibility of DNA Devices Observed by Small-Angle X-ray Scattering. *Nano Lett.* 2016, *16*, 4871–4879. Copyright (2016) American Chemical Society.

Conformational changes, flexibility, and self-assembly [71,72] processes of DNA nanostructures are being investigated using SAS techniques with increased frequency. The structural features determined by these solution-based techniques offer structural insights that are distinct from those provided by techniques that require the nanostructure to be fixed onto a substrate. For example, an X-shaped DNA-based molecular switch has been examined through SAXS, solution fluorescence resonance energy transfer (FRET), single-molecule FRET, and transmission electron microscopy (TEM) to determine the population of the two distinct conformational states (Figure 4). The switch’s conformation, which closes to form a linear rod-like structure in high ionic strength environments, was shown through SAXS and solution FRET to have a statistically significantly lower population of molecules in the linear conformation than was determined by single molecule FRET or TEM. It is suggested that the fixation to surface, dyeing, and/or the manual assignment of conformations of TEM images may bias these experimental methods towards a closed conformation, while the solution-based techniques gave more accurate assessments of the particle conformations [72]. The increased availability of SAS instruments located off high flux, synchrotron sources allowed these measurements to be made with reasonable signal to noise profiles on samples at concentrations of only 25–100 nM. They have been able to detect conformational changes triggered by changes in the solution environment. These studies were followed up with time-resolved SAXS [74] to monitor this large-scale conformational transition and it was found that it switches from its open to closed conformation upon addition of MgCl_2_ within milliseconds, which is close to the theoretical diffusive speed limit. The construction of functional NANPs will likely require dynamic structures that can undergo controllable conformational changes and SAXS is well placed as a tool for resultant structural kinetic studies. DNA devices based on shape complementary stacking interactions have been demonstrated to undergo reversible conformational changes triggered by changes in ionic environments or temperature. In another, unrelated experiment, molecular dynamics and SANS were used in combination to predict and test the gelling properties of tetravalent DNA nanostars as a function of temperature [71]. The time-resolved growth of the DNA nanostar gel was monitored by following changes in a signature peak intensity, and, thus, these studies allowed for kinetic and thermodynamic measurements of the nanostar structural formation.

**Figure 5:**
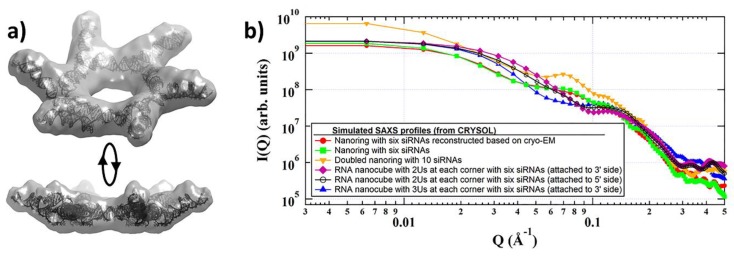
(**a**) Cryogenic-electron microscopy (cryo-EM) reconstruction of RNA nanoring functionalized with six short interfering RNAs (siRNAs) and (**b**) its calculated SAXS profile (red circles) compared to the calculated SAXS profile for other predicted NA nanoparticle structures. Profiles were calculated from models based on cryo-EM pdb files using the program CRYSOL [73].

*SAS Vision Application for Nucleic Acid Architectures:* One of the examples where SAS can be readily employed may be seen in the recent achievements of RNA nanotechnology, where two orthogonal NANP designing strategies (exemplified by RNA nanocubes [4,8,12,13,23,25,75] and RNA nanorings [7,13,24,41,75,76,77]) were introduced, with potential for broad use in nanotechnology and biomedical applications. In one strategy (nanocubes) the RNA strands are specifically designed to only form intermolecular bondings with their cognate partners while avoiding the formations of any intramolecular secondary structures. Another strategy (nanorings), takes advantage of RNA long-range tertiary interacting motifs that require the formation of specific secondary structures of individual monomers, and the intermolecular interactions are activated in the presence of magnesium ions. Both NANPS were tested against several different cancer- and HIV-infected cell lines and showed a significant therapeutic effect. Furthermore, the desired activity of these functional NANPs was demonstrated remarkably in vivo [7]. Importantly, the immunorecognition of NANPs by human peripheral blood mononuclear cells strongly depends on the type of NANPs and the extent of their functionalization with siRNAs [13,78,79]. In vitro characterization with cryo-EM microscopy has revealed that the structure of the nanoring functionalized with six siRNAs has a pinwheel-like crown shape (Figure 5a). That topology, if accurately reflecting the in vivo state, may affect the efficiency of the intracellular release of siRNAs through ‘dicing’, due to steric issues, and influence their interactions with the pathogen recognition receptors of the immune system. The issues of imprecisely predicted and verified topology may become even more evident in the case of 3D polyhedral self-assembled functional nanostructures such as nanocubes [4]. Figure 5b provides the calculated SAXS profiles for several of these predicted structures, demonstrating that SAXS data is sufficient to differentiate between the various architectures. It is possible that further optimization of the existing designs is needed (such as an extension of dicable siRNA-containing arms, modification of the 5′-end of the scaffold, changes in base composition, or introduction of additional RNA structural motifs, etc.) to ensure the enhancement of siRNA release and processivity.

Moreover, 3D structures of other individual, relatively bulky groups, such as aptamers or antibodies introduced for targeting, are not known, and their function may be attenuated due to steric clashes within NANPs. Therefore, alternative approaches that can provide complementary data about 3D orientations and shapes of the NANPs and their individual components in solution are needed. Utilizing natural contrast between the scattering components in these systems, neutron scattering will allow for determination of the structural parameters of the NANPs bound to any functional groups. Another vision for the use of SAS in structural characterization of NANPs is to extend the SAS profile collected to include a larger angle scattering region (WAXS). These data may be useful for investigating the Ag-Ag distances within DNA-based assemblies of fluorescent silver nanoclusters [80].

Additionally, delivery of NANP-based therapeutics in vivo is one of the most challenging tasks due to RNA’s negative charge, chemical instability, and stimulation of immune system responses. Investigating different potential carriers, such as lipids or cell-penetrating peptides, for in vivo delivery of RNA therapeutics is therefore one area of RNA nanotechnology that would benefit from SAS-based approaches. Experiments with NANPs employing various carriers such as magnetic nanoparticles [81], lipids [82], mesoporous silica-based nanoparticles [79], polysilsesquioxane [83], and bolaamphiphiles or ‘bolas’ [84,85] have already been successfully initiated. The use of SANS can significantly improve our current understanding of the interactions between the NANPs and carriers, which can further improve NANP delivery in vivo. For example, bolas consist of positively charged acetylcholine head groups on each side of a hydrophobic chain. In aqueous solution, these bolas form micelles and are efficiently associated with siRNAs for their further delivery in vivo. It was also recently demonstrated that bolas can form vesicles, rather than micelles, and can be used for delivery of encapsulated analgesic peptides and small molecules within a mouse brain [86,87]. These vesicles may become strong candidates for the delivery of functional RNA nanoparticles in vivo, especially across the blood-brain barrier to glioblastomas. Preliminary, unpublished results indicate formation of stable siRNA/bola vesicle complexes and cryo-EM images show changes in the shape of the particle upon siRNA addition. Constraints for the formation of functional RNA nanoparticles/bola vesicle complexes must be directed by the architecture of the components including shape, size, and total charge. Therefore, comparison of different shapes for functional RNA nanoparticles and different RNA-to-bola ratios will be necessary to maximize the RNA-bola interaction capacity. The self-assembly of similar, stable monomolecular nucleic acid lipid particles has been studied by SAXS and complemented by SANS and TEM [88]. These SAXS data confirmed the overall size and spherical shape of the particles, whereas the inherent contrast between nucleic acid and lipid moieties’ neutron scattering allowed for a more detailed structural description of the core shell-like structure of these particles.

## 3. Conclusions

Small-angle X-ray and neutron scattering are structural characterization techniques that are well placed to determine the conformation of nucleic acid nanoparticles under varying solution conditions, thus allowing for optimization of their design. SAS results should complement and extend the structural information obtained through direct imaging techniques and other high-resolution structures. SAS experiments provide information on the overall shapes and particle dimensions of macromolecules and are ideal for following conformational changes and dynamics of the molecular ensemble as it behaves in solution. In addition, the inherent differences in the neutron scattering of nucleic acids, lipids, and proteins, as well as the different neutron scattering properties of the isotopes of hydrogen, combined with the ability to uniformly label biological macromolecules with deuterium, allow for the characterization of the conformations and relative dispositions of the individual components within an assembly of biomolecules.

## Figures and Tables

**Table 1:** Comparison of nanoparticle structural characterization techniques.

Technique	Parameters Analyzed/Advantages	Limitations
**Solid State/Static Techniques**		
Crystallography [28]	High resolution molecular structure Broad Mass range Model building is well-developed	Static crystalline state structure; may not reflect dynamic or flexible structures Sample must form a crystal
Scanning Electron Microscopy (SEM) [28,29]	Particle size, size distributions, shape Sample preparation is relatively simple Structure in native state Allows analysis of hydrated materials without fixation, drying, freezing, or coating	Limited to larger molecules (up to ~200 nm) Highly dependent on electron microscopy (EM) techniques and access to costly equipment Cannot be used on certain biological materials due to degradation caused by the electron beam Low resolution
Transmission Electron Microscopy (TEM) [28]	Particle size, size distributions, shape Produces high resolution images that can provide information about structure and elemental composition High resolution TEM has Å resolution	Harsh chemical treatment of the sample Statistics are highly dependent on technique 2D images Samples need to be dehydrated, collected on metal mesh, and stained Small viewing section of sample
Atomic Force Microscopy (AFM) [30,31]	Provides a three-dimensional surface profile Minimal sample preparation Shown to give true atomic resolution in ultra-high vacuum (UHV) and, more recently, in liquid environments	Can only image a maximum height on the order of 10–20 micrometers and a maximum scanning area of about 150 × 150 micrometers Images can also be affected by hysteresis of the piezoelectric material Possibility of image artifacts Must immobilize the sample onto a substrate
**Solution State/Native Techniques**		
Static Light Scattering (SLS)/Dynamic Light Scattering (DLS)/ Zeta Potential [28]	Hydrodynamic particle size, size distributions, surface charge Sample volumes are small (μL) Particle size across a broad range (~0.1 nm to ~10 µm) Allows measurements under physiological conditions	Can only measure solid particles, polymers, and proteins dispersed in a solvent or emulsions Light absorption by the dispersant or sample can interfere with detection Concentration dependent Samples need to be homogenous Little shape information; size of particles can be under or over-estimated Dust/traces of agglomerates can interfere with results Cannot distinguish between similarly sized populations without coupling to a separation
Nuclear Magnetic Resonance (NMR) [32]	High resolution structure 3D structure in solution Dynamics can be studied	High sample purity and concentration required Computational simulation is challenging Sample MWs typically limited to below 40–50 kDa Water soluble samples
Small-Angle X-ray Scattering (SAXS) [28,33]	Structure in native state Particle size and shape, size distribution, particle interactions and interatomic distances: some parameters determined with sub Å precision Small sample size (10–25 μL solution; 0.01–10 mg/mL) Highflux synchrotron sources allow for time-resolved, kinetic studies	Low-resolution shape information interpreted from interatomic distance distributions Highest level of structural information requires pure, monodisperse samples
Small-Angle Neutron Scattering (SANS) [28,33]	Amenable to contrast variation Sensitive to fluctuations in the nuclei density of the sample	Experiments require access to user facilities with appropriate neutron source and instrumentation Flux of neutron source is intrinsically low
